# Eosinophilic meningitis in New Caledonia: The role of *Angiostrongylus cantonensis*?

**DOI:** 10.1371/journal.pone.0254964

**Published:** 2021-08-12

**Authors:** Bénédicte Melot, Gauthier Delvallez, Ann-Claire Gourinat, Nicolas Molko, Cyrille Goarant, Yves-Marie Ducrot, Emilie Huguon, Cécile Cazorla, Martine Chauvet, Antoine Biron, Julien Colot

**Affiliations:** 1 INSERM, UMRS 1142 LIMICS, Sorbonne Université, Paris, France; 2 Experimental Bacteriology Group, Pasteur Institute in New Caledonia, New Caledonia, France; 3 Microbiology Laboratory, Territorial Hospital of Noumea, New Caledonia, France; 4 Department of Neurology, Territorial Hospital of Noumea, New Caledonia, France; 5 Lifou Health Center, New Caledonia, France; 6 Department of Pediatrics, Territorial Hospital of Noumea, New Caledonia, France; 7 Department of Infectious Diseases, Territorial Hospital of Noumea, New Caledonia, France; Tulane University, UNITED STATES

## Abstract

**Introduction:**

Eosinophilic meningitis is a rare form of meningitis with sequelae or death occurring in approximately 2–3% of cases. The most frequent etiological agent is the parasite *Angiostrongylus cantonensis*. The aim of this study was to characterize New Caledonian cases and to assess the extent to which of *A*. *cantonensis* was involved.

**Material and methods:**

We performed a retrospective study of all cases of eosinophilic meningitis (EM) admitted to the Territorial Hospital of New Caledonia, from 2004 to 2019. We performed a descriptive and a multivariate analysis to identify association of variables with severe and fatal cases (or cases with sequelae).

**Conclusion:**

Angiostrongyliasis was confirmed as being responsible for 17 of the 92 reported EM cases in New Caledonia from 2004 to 2019 with most being young adults and non-walking infants, and with two peaks of incidence one during the dry season and one during the rainy season. Considering the high incidence and regularity of cases, the potential reservoirs should be identified to target prevention campaigns.

## Introduction

Eosinophilic meningitis (EM) is defined as the presence of more than 10 eosinophils/mm^3^ in the cerebrospinal fluid (CSF) and/or eosinophils accounting for more than 10% of CSF leukocytes [1]. Invasion of the central nervous system by helminth parasites is the most common cause, but other infections as well as several non-infectious etiologies have also been identified [2].

*Angiostrongylus cantonensis*, the rat lungworm, is the most frequent parasitic cause of EM [3]. EM, in this case, results from ingestion of or possible [4] contact with intermediate (snails or slugs) or paratenic hosts infected by third-stage larvae followed by larval migration within the nervous system.

From 1944 to 2008, more than 2,800 human cases of EM due to *A*. *cantonensis* had been reported in the world [5], especially in southeast Asia (China [6–8], Thailand [9], Vietnam [10] India [11]), and Oceania particularly Hawaii [12], French Polynesia [13, 14], American Samoa [15], and Papua New Guinea [16] as well as in Brazil [17], the US [18] and the Caribbean [19]. No doubt there have been many more cases since 2008.

The first identification of worms in the CSF of a patient with EM occurred in Taiwan in 1945 but the connection to the boy’s illness was not made at that time [20]. Since 1961, there have been numerous investigations into the epidemiology of the disease [21]. The predominance of cases in the Pacific and southeast Asia is due in part to ship-borne dissemination of infected rats and snails in this area during World War II [22]. There were outbreaks from 1948 to 1957 in the Pacific, including one in 1951 in New Caledonia [23] and since then, regular cases in both adults and children. New Caledonia is an overseas collectivity of France in the southwestern Pacific Ocean, with a population of 271,000 inhabitants in 2019, of whom 75% live in the southern province [24]. The archipelago has a tropical maritime climate. There are two seasons: a hot and rainy season from November to April and a dry and cooler season from May to October. Although two-thirds of the population live in the capital metropolitan area, many people live in rural areas where cultural habits include farming and frequent consumption of seafood and raw fish [24]. In New Caledonia, the vectors of *A*. *cantonensis* transmission remain unclear, although the giant African snail, *Lissachatina fulica*, has been incriminated as in other locations in the Indian and Pacific Oceans [25, 26]. Between 1965 and 1967, Ash [27] studied various slugs and planarians in the territory and suggested that the most probable route of infection in New Caledonia was through accidental ingestion of infected planarians on raw vegetables. The objective of our study was to describe the recent New Caledonian EM cases in order to guide the implementation of appropriate diagnostic tests in this endemic area and improve prevention measures for the population and caregivers.

## Material and methods

We retrospectively studied all cases of EM admitted to the Territorial Hospital of New Caledonia, from October 2004 to October 2019. This hospital, located in Noumea the administrative capital, has 645 beds and is the referral center for the whole territory. Cases were defined based on the association of any neurological symptoms compatible with EM together with biological EM (presence of CSF pleocytosis above 10 eosinophils/mm^3^ or ≥ 10% eosinophils of the total CSF leukocyte count). Regarding ethical consideration, all data analyzed were collected as part of routine diagnosis and treatment and patients were diagnosed and treated according to national guidelines and standard protocols. No experimental or new protocols were used for testing blood and CSF. All data were anonymously reported in a standardized case report form and entered in an Excel database (Microsoft, Seattle, WA). The French National Commission Information Science and Liberties Commission authorizes the retrospective use of anonymous data already collected in patient files [28]. The data used were completely anonymous, i.e. without an identification number.

We collected data for the following variables based on medical files: age, gender, ethnic origin, medical history, clinical presentation (incubation time, symptoms, disease course), biological results (blood and CSF cell count, CSF pressure, serology, *A*. *cantonensis*-PCR, parasitological examination of stool, natremia (hyponatremia was defined as natremia lower than 135 mmol/L)), fundoscopic examination, electroencephalogram, electromyogram, imaging (CT scan, MRI), type of treatment, length of hospital stay, and outcome at 1 year post diagnosis.

Severe cases were defined as patients who had either encephalitis and/or neurological deficit including cranial nerve involvement [29–33]. Fatal cases and cases with sequelae were defined as patients who had neurological sequelae, recurrences or had died after one year of follow up with no other identified cause of death.

All serological tests performed during the study were sent to the New South Wales (NSW) Health Pathology laboratory, Australia. Because of its high cost, serology was not performed on all patients. This test was performed with serum or CSF. There is no commercial kit for it. The NSW Health Pathology laboratory uses a crude adult worm extract to coat our ELISA plates. Thus, the test does cross-react with other helminth infections. It can detect antibodies to the worm antigen from CSF but there is a delayed reaction of about >3–4 weeks. The NSW laboratory estimates the specificity of this test to be 70% (Rogan Lee, personal communication, 17 mars 2021). Sensitivity is not yet determined and would be low because there is a lag between neurological symptoms and detection of antibodies in serum or CSF. Other inhouse ELISA estimate sensitivity between 83 and 97% and specificity between 80 and 100% [34]. Early CSF and serum samples will give a false negative result. Early detection of eosinophils in the CSF and peripheral blood smears may be the early indications of neuroangiostrongyliasis. In the case of patients clinically suspected for neuroangiostrongyliasis, a repeat serologic test three weeks after onset of illness is recommended.

PCR for *A*. *cantonensis* became available in January 2014 in our laboratory. In a series published in 2016, its sensitivity was 90% and specificity 100% [35].

Total DNA from human CSF (200 μl) was prepared using the MagNA Pure LC total nucleic acid isolation high performance Kit (Roche Diagnostics) with a 50 μl elution volume. TaqMan PCR amplification was performed in a 20 μL total volume containing Light cycler 480 probes master (Roche Diagnostic) with 0.3 μM of the species-specific primers and probe targeting the internal transcribed spacer (ITS1) region described by Qvarnstrom et al [36], AcanITS1F1 (5’TTCATGGATGGCGAACTGATAG-3’) and AcanITS1R1 (5’-GCGCCCATTGAAACATTATACTT-3’), and 0.1 μM probe AcanITS1P1 (5’-6-FAM-ATCGCATATCTACTATACGCATGTGACACCTG-BHQ1-3’ and 4μL of the extracted DNA specimen. The PCR conditions were 95°C for 10 min followed by 45 cycles of 95°C for 15 sec and 60°C for 60 sec. The positive control was an ITS1 plasmid kindly provided by the US Centers for Diseases Control and Prevention, confirmed by sequencing. The negative control was PCR-grade ultrapure water. The efficiency of DNA extraction and the possible presence of inhibitors in the samples were tested using the following primers and probe targeting a fragment of the human RNase3P using the same PCR conditions: RNAseP3F (5’-CCAAGTGTGAGGGCTGAAAAG-3’), RNAseP3R (5’-TGTTGTGGCTGATGAACTATAAAAGG-3’), and RNAseP3Pr: (5’-VIC-CCCCAGTCTCTGTCAGCACTCCCTTC-BHQ-1-3’).

We first completed a descriptive analysis of our cases, calculating mean with standard deviation (SD), median with interquartile ranges (IQR) were calculated for continuous variables, and percentages for categorical variables. Because of the small sample size, some biological variables were dichotomized. We analyzed each variable by comparing data from severe cases with data from mild cases using Chi squared or Fisher’s exact tests for categorical variables and with Students’ t-tests for continuous variables. We also used multivariate analysis of variance to identify variables associated with severe cases and with fatal cases or cases with sequelae adjusted for the other variables. For the multivariate analysis we kept all variables from the single analysis that had a p value < 0.2 in the univariate analysis. The final model was tested using the Hosmer and Lemeshow test. The p value was considered significant when < 0.05. All analyses were done with R VERSION 3.5.0.

Finally, we did a subgroup analysis of all the cases confirmed as being caused by *A*. *cantonensis* with a descriptive analysis of this group and a multivariate analysis of variance to identify variables associated with fatal cases or cases with sequelae in this subgroup adjusted to the other variables.

## Results

**Fig 1:**
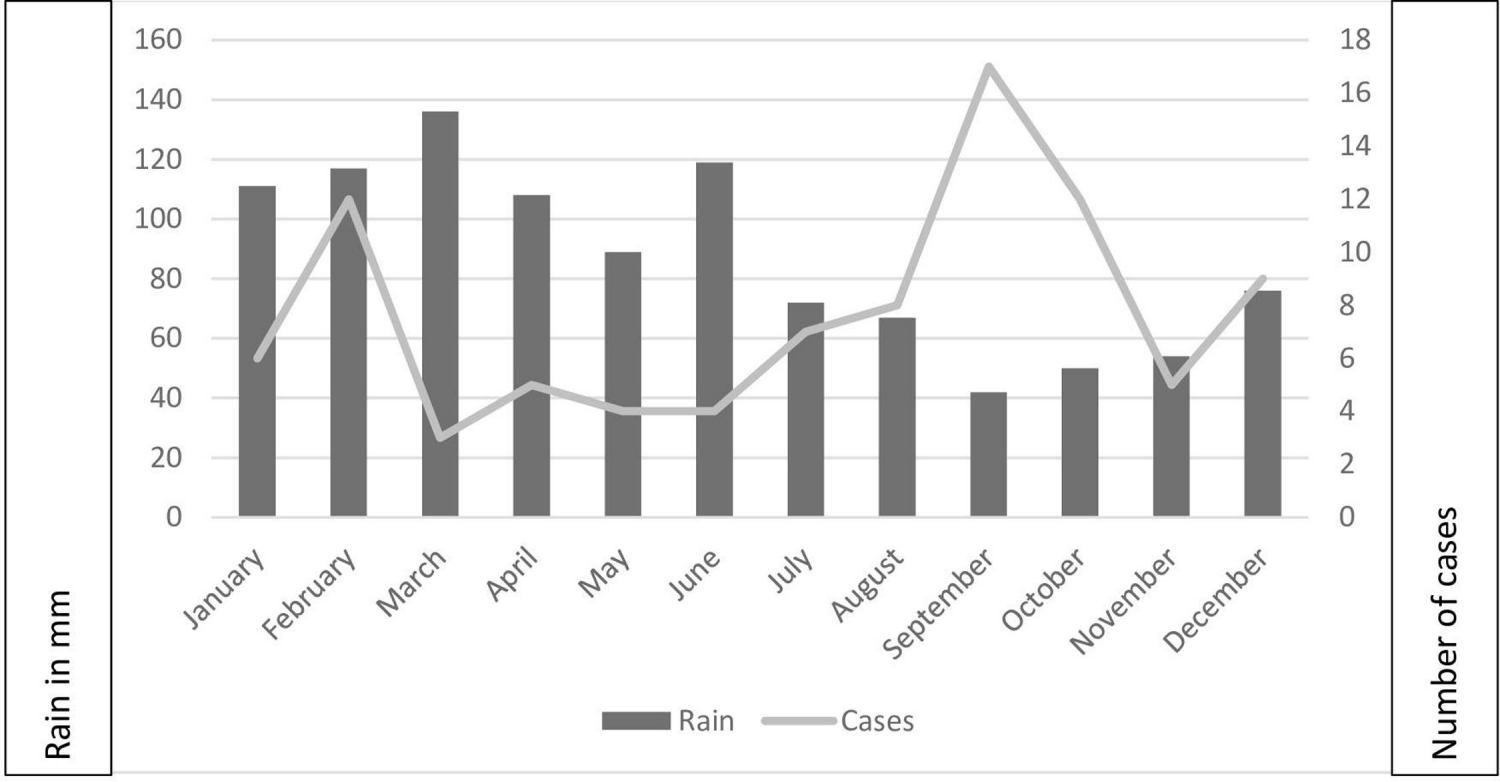
Monthly distribution of the New-Caledonian cases according to average rainfall in mm in Noumea, from 2004 to 2019, n = 92.

We identified 92 cases of EM between 2004 and 2019 corresponding to an average of 5.8 cases per year (SD = 1.41) unevenly distributed: significantly more cases were observed in 2010 (9 cases) and 2011 (13 cases) than in other years (p<0.01). The mean age was 26 including 22 patients under 5, and 5 over 65. The most affected age group was young children between 0 and 4 years old (23.9%) with an annual incidence almost four times higher than the other age categories (1.06 vs 0.28/1000) and cases were predominantly among Melanesians (82.6%) for which the annual incidence was 0.045 per 1000 inhabitants per year with a relative risk of 6.42 times that for Europeans living in New Caledonia (0.007 cases per 1000 inhabitants per year) (Table 1). The mean number of cases per month was 7.66 (SD = 1.17); there were two periods with significantly more cases (p<0.01), one in February with 17 cases (during the rainy hot season) and one in September/October with 12 cases each (during the dry cold season) **(Fig 1).**

**Table 1:** Socio-demographical features of New Caledonian cases of EM from 2004 to 2019 according to severity, n = 92.

Variables	Total		Severe		Non severe		Crude OR*	95% CI**	P value
	N = 92	%	N = 29	%	N = 63	%			
Age							1.03	1.01–1.06	0.005
Mean (SD)	25		34.2 (22.7)		20.7 (18)				
Median (IQR)	22.5		36 (27)		15 (30.5)				
Age class (years old)									0.03
0–4	22	23.9	5	17.2	17	27			
5–18	19	20.7	2	6.9	17	27	0.4	0.07–2.35	0.31
19–25	7	7.6	3	10.3	4	6.3	2.55	0.4–15.4	0.3
>25	44	47.8	19	65.5	25	39.7	2.58	0.8–8.26	0.11
Sex masculine							0.94	0.38–2.33	0.9
No	34	37.0	11	37.9	23	36.5			
Yes	58	63.0	8	62.1	40	63.5			
Melanesian							1.02	0.32–3.25	0.98
No	16	17.4	5	17.2	11	17.5			
Yes	76	82.6	24	82.8	52	82.5			
Rural							1.0	0.33–2.9	0.99
No	19	20.7	6	20.7	13	20.6			
Yes	73	79.3	23	79.3	50	79.4			
Province									0.05
Islands	12	13.0	2	6.9	10	15.9	0.12	0.01–0.97	
North	28	30.4	12	41.4	16	25.4	0.45	0.09–2.26	
South	44	47.8	10	34.5	34	54.0	0.18	0.04–0.87	
Others (Vanuatu, Wallis)	8	8.7	5	17.2	3	4.8			
Seasonality							0.92	0.38–2.24	0.85
May-October	52	56.5	16	55.2	36	57.1			
November-April	40	43.5	13	44.8	27	42.9			

* OR = odds ratio.

** CI = confidence interval.

The sex ratio was 1.7 M:F (58 males, 34 females). The majority of the patients lived in rural areas (79.3%) (Table 1).

**Fig 2:**
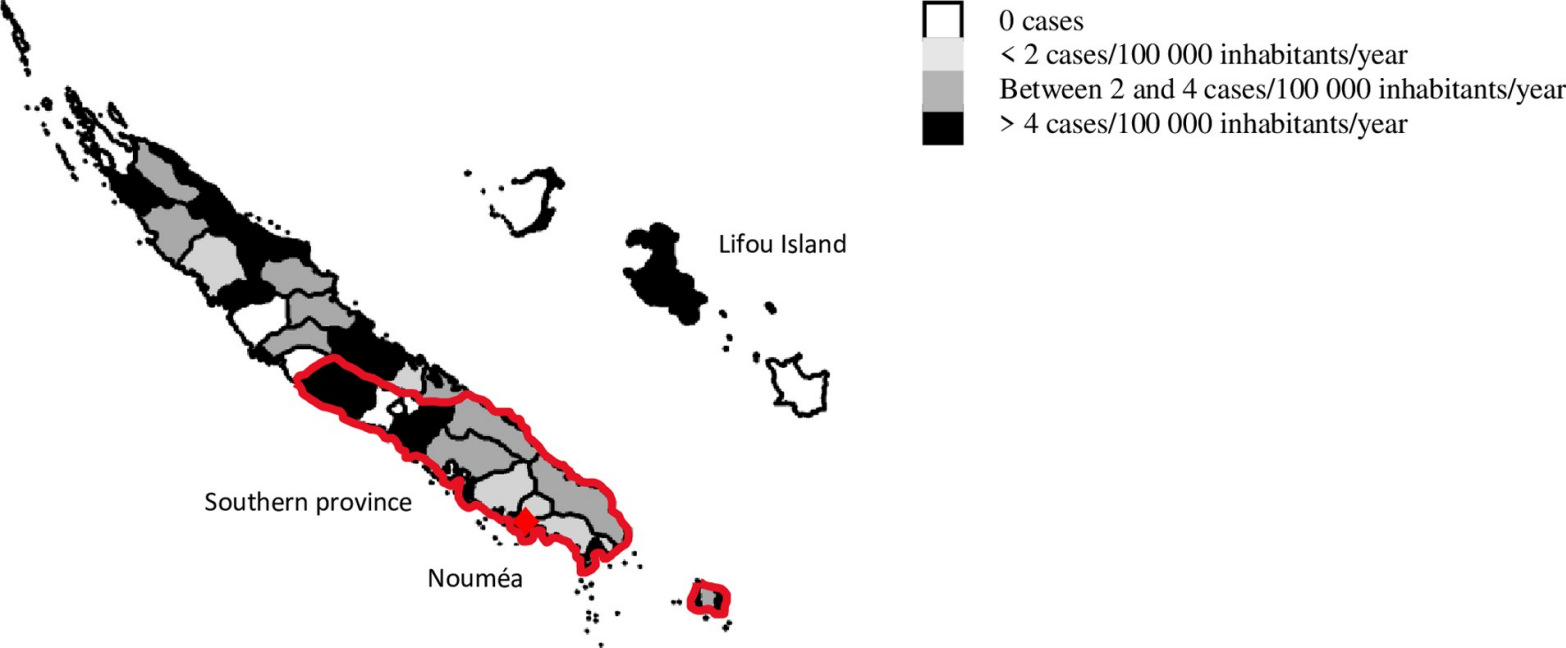
Map of the incidence of cases of EM in New Caledonia from 2004 to 2019, n = 92.

There was no statistically significant difference in presence or absence of sequelae or severity according to sex ratio, ethnic origin, or way of life (rural or urban). The number of cases observed in the southern province was higher than in the other provinces but the incidence (number of cases per 1000 population) was higher in the northern humid regions and on the Loyalty islands; 5 cases were from Vanuatu, 1 from Wallis and 1 from France but had travelled to Vanuatu (**Fig 2**).

Lifou (one of the Loyalty Islands within the New Caledonian archipelago) was the island with the most cases: 9 of the 12 identified on the Loyalty Islands (the 3 others were identified on Ouvea). In 2018 and 2019, there were more cases on Lifou Island (4 cases from 2004 to 2015 and 5 cases in 18 months in 2018 and 2019) although these are too few cases to test for a statistically significant increase.

The average duration of hospital stay was 8.8 days (range 0–56). Seven patients were admitted to the ICU. The average duration of symptoms was 12.7 days (range 0–130).

The most common symptoms were headache (55 cases), vomiting (36), fever (26), deterioration of general condition defined by anorexia, asthenia or loss of weight (25 cases), and neck stiffness (22). Almost one third of cases were severe (29), defined by encephalitis (11), or radiculitis (18 including cranial nerves). Among patients with convulsions (10), the majority had encephalitis (6) (**Table 2**).

**Table 2:** Clinical features of New Caledonian cases of EM from 2004 to 2019 according to severity, n = 92.

Variables	Total		Severe		Not severe		Crude OR	95% CI	P value
	N = 92	%	N = 29	%	N = 63	%			
Duration of symptoms (d)							2.23	1.5–2.9	0.02
Mean (SD)	12.73		20.7(28.1)		8.9(7.7)				
Median (IQR)	7		15(14)		6.25(8.9)				
Duration of hospitalization (d)							1.1	1.03–1.19	0.009
Mean (SD)	8.8		16.2 (28.6)		5.4 (4.2)				
Median (IQR)	5		8 (10)		4(4.5)				
ICU							1.7	0.23–10.7	0.6
No	85	92.4	26	89.7	59	93.7			
Yes	7	7.6	3	10.3	4	6.3			
Headache							0.93	0.38–2.28	0.87
No	37	40.2	12	41.4	25	39.7			
Yes	55	59.8	17	58.6	38	60.3			
Fever							1.96	0.76–5.05	0.16
No	66	71.7	18	62.1	48	76.2			
Yes	26	28.3	11	37.9	15	23.8			
Crying							0.53	0.06–4.9	0.57
No	87	94.6	28	96.6	59	93.7			
Yes	5	5.4	1	3.4	4	6.3			
Deterioration of general condition							0.6	0.2–1.7	0.34
No	67	72.8	23	79.3	44	69.8			
Yes	25	27.2	6	20.7	19	30.2			
Grey complexion							1.09	0.09–12.5	0.9
No	89	96.7	28	96.6	61	96.8			
Yes	3	3.3	1	3.4	2	3.2			
Coma							4.6	0.4–53	0.22
No	89	95.7	27	93.1	62	98.4			
Yes	3	3.3	2	6.9	1	1.6			
Drowsiness							0	0.-inf	1
No	88	95.7	29	100.0	59	93.7			
Yes	4	4.3	0	0.0	4	6.3			
Vomiting							0.37	0.14–1	0.05
No	56	60.9	22	75.9	34	54.0			
Yes	36	39.1	7	24.1	29	46.0			
Stiff neck							0.16	0.03–0.74	0.02
No	70	76.1	27	93.1	43	68.3			
Yes	22	23.9	2	6.9	20	6.9			
Convulsion							3.8	1–15	0.05
No	82	89.1	23	79.3	59	93.7			
Yes	10	10.9	6	20.7	4	6.3			

Cerebrospinal fluid (CSF) analysis showed on average 558 white blood cells per mm^3^ (WBC/mm^3^) (range 7–4000), with an average of 37.3% being eosinophils. CSF eosinophil counts were higher than 40% in 37 of the cases among which 24 had neither biological confirmation of *A*. *cantonensis* nor an alternative diagnosis. To establish the etiology of EM, a biological test for *A*. *cantonensis* was performed in 35 cases: 14 patients had only a serology test, in serum and/or CSF, 20 had only a PCR test in CSF and one patient had both tests (Table 3). The diagnosis of angiostrongyliasis was confirmed in 17 cases: of the 15 serology tests performed, 2 were positive in serum, 4 in the CSF and serum; of the 21 PCRs performed 11 were positive (Table 3). In addition, we found in CSF a mean protein level of 0.92g/L, but greater than 1.0 in 27 cases, and the average glucose level was 2.73 mmol/L. The CSF pressure was heightened (higher than 200 mm H_2_0) in 9 cases including 3 with radiculitis. Blood analysis showed an average level of eosinophils of 1762 per mm^3^ (range 0–5470); hyponatremia was noted in 17 cases.

**Table 3:** Biological features of the New Caledonian cases of EM from 2004 to 2019 according to severity, n = 92.

Variables	Total		Severe		Not severe		Crude OR	95% CI	P value
	N = 92	%	N = 29	%	N = 63	%			
% CSF eosinophils							1	0.99–1.03	0.42
Mean (SD)	37.3		40.2 (24.3)		36.0 (22.7)				
Median (IQR)	32		36 (36)		31(36)				
CSF eosinophils >40%							1.32	0.54–3.22	0.37
No	55	60.0	16	55.2	39	61.9			
Yes	37	40.0	13	44.8	24	38.1			
CSF GB (/mm^3^)							1	0.99–1	0.4
Mean (SD)	558		486 (330)		591 (638)				
Median (IQR)	480		399 (440)		500 (782)				
CSF proteins (g/L)							2	0.97–4.15	0.06
Mean (SD)	0.92		1.2 (1.2)		0.8(0.45)				
Median (IQR)	0.76		0.87 (0.71)		0.72 (0.52)				
CSF proteins > 1 g							1.8	0.7–4.6	0.22
No	65	70.7	18	62.1	47	74.6			
Yes	27	29.3	11	37.9	16	25.4			
CSF glucose (mmol/L)							1.58	0.92–2.72	0.1
Mean (SD)	2.73		2.9(1.04)		2.63 (0.72)				
Median (IQR)	2.66		2.8 (1.15)		2.6 (0.92)				
Blood eosinophils (/mm^3^)							1	0.99–1	0.68
Mean (SD)	1762		1853 (1565)		1719 (1437)				
Median (IQR)	1270		1230 (2100)		1280 (2070)				
% blood eosinophils							1.02	0.97–1.07	0.54
Mean (SD)	15.11		17 (12.8)		14.2 (9.6)				
Median (IQR)	14.3		14.9 (15.1)		13.7 (15.3)				
Elevated CSF pressure							3.07	0.76–12	0.11
No	83	90.2	24	82.8	59	93.7			
Yes	9	9.8	5	17.2	4	6.3			
Positive serology									0.6
No	9	9.8	3	10.3	6	9.5			
Yes	6	6.5	3	10.3	3	4.8	2	0.24–16.6	0.5
Not performed	77	83.7	23	79.3	54	85.7	1.78	0.2–3.7	0.8
Positive CSF serology									0.11
No	7	7.6	1	3.4	6	9.5			
Yes	4	4.3	3	10.3	1	1.6	18	0.81–400	0.07
Not performed	81	88.0	25	86.2	56	88.9	2.7	0.3–23.4	0.37
Positive CSF PCR							2.67		0.35
No	10	10.9	2	6.9	8	12.7			
Yes	11	12	2	6.9	9	14.3	0.89	0.1–7.8	0.91
Not Performed	71	77.2	25	86.2	46	73.0	2.1	0.43–11	0.35
Hyponatremia							0.81	0.24–3.01	0.77
No	75	81.6	23	79.3	52	82.5			
Yes	17	18.4	6	20.7	11	17.5			

Taken together, *A*. *cantonensis* proved to be a major cause of EM since it was confirmed in 17/35 (49%) of patients who were biologically investigated. An alternative diagnosis was identified in 11 cases: in 5 cases a serology for toxocariasis was positive, in 6 cases a bacteriological cause was identified from CSF: 3 with *Mycobacterium tuberculosis*, one with *Nocardia* sp, one with leptospirosis and one with *Streptococcus pyogenes*.

Radiological and other paraclinical results are presented in Table 4. A brain CT scan was abnormal in 9 cases (edema). An MRI was abnormal in 5 cases (diffuse hyperintensity especially in Flair and diffusion sequences). An electroencephalography (EEG) was abnormal in 10 cases (slow waves). An electromyography (EMG) was done in 2 cases and was normal. Fundoscopic examination was done in 12 cases and was abnormal in 2 cases (papillary edema) (**Table 4**).

**Table 4:** Radiological and other paraclinical features of New Caledonian cases of EM from 2004 to 2019 according to severity, n = 92.

Variables	Total		Severe		Not severe		CrudeOR	95% CI	P value
	N = 92	%	N = 29	%	N = 63	%			
Abnormal CT scan							1.1	0.25–4.7	0.9
No	83	90.2	26	89.7	57	90.5			
Yes	9	9.8	3	10.3	6	9.5			
Abnormal MRI							0	0-inf	1
No	87	94.6	23	82.8	63	100.0			
Yes	5	5.4	5	17.2	0	0.0			
Abnormal EEG							1.52	0.39–5.8	0.54
No	82	89.1	25	86.2	57	90.5			
Yes	10	10.9	4	13.8	6	9.5			
Abnormal fundoscopic examination							0	0-inf	1
No	90	97.8	29	100.0	61	96.8			
Yes	2	2.2	0	0.0	2	3.2			

Steroids were given in 14 cases to reduce inflammation for an average duration of 26.6 days, the main indication being encephalitis (6 cases). Significantly more patients who died or experienced sequelae had received steroids (Crude OR = 10.2, CI = 2.9–36, p < 0.001). Antiparasitic treatments were given in some cases: albendazole was given in 27 cases for an average of 4 days, and ivermectin was given in 5 cases for an average of 3 days. Therapeutic lumbar puncture to reduce CSF pressure was done in only 2 cases. Antibiotics were given in 12 cases with no differences in severity and no impact on sequelae (**Tables 5 and 6**).

**Table 5:** Treatment of New Caledonian cases of EM according to severity, n = 92.

Variables	Total		Severe		Not severe		Crude OR	95% CI	P value
	N = 92	%	N = 29	%	N = 63	%			
Albendazole							1.1	0.43–2.93	0.81
No	65	70.07	20	69.0	45	71.4			
Yes	27	29.3	9	31.0	18	28.6			
Duration of albendazole (d)									0.39
Mean	3.9		6 (7.3)		3.2 (1.1)				
Median	3		3 (0)		3 (0)				
Ivermectin							0.53	0.06–4.93	0.57
No	87	94.6	28	96.6	59	93.7			
Yes	5	5.4	1	3.4	4	6.3			
Duration of ivermectin (d)									0.7
Mean	2.8		2(0)		3 (2.8)				
Median	2		2(0)		2 (3)				
Steroids							2.55	0.8–8.1	0.11
No	78	84.8	22	75.9	56	88.9			
Yes	14	15.2	7	24.1	7	11.1			
Duration of steroids (d)							1.6		0.11
Mean	26.6		41 (34)		11 (11)				
Median	15		30 (45)		9 (12)				
Antibiotics							1.1	0.3–4	0.88
No	80	87.0	25	86.2	55	87.3			
Yes	12	13.0	4	13.8	8	12.7			

**Table 6:** Final model of New Caledonian cases of EM according to death/sequelae, n = 92.

Variables	Total		Death/sequelae		Without sequelae		Adjusted OR	95% CI	P value
	N = 92	%	N = 29	%	N = 63	%			
Encephalitis							1.27	0.7–1.25	0.87
No	81	88.0	13	76.5	68	90.7			
Yes	11	12.0	4	23.5	7	9.3			
Radiculitis							1.2	0.94–1.5	0.13
No	74	80.4	10	58.8	64	85.3			
Yes	18	19.6	7	41.2	11	14.7			
Cranial radiculitis							1.18	0.87–1.6	0.26
No	81	88	11	64.7	70	93.3			
Yes	11	12	6	35.3	5	6.7			
Positive serology in CSF							0.8	0.48–1.3	0.36
No	7	7.6	1	5.9	6	8.0			
Yes	4	4.3	2	11.8	2	2.7			
Not performed	81	88.0	14	82.4	67	89.3			
Modified MRI							1.5	1–2.3	0.05
No	87	94.6	13	76.5	74	98.7			
Yes	5	5.4	4	23.5	1	1.3			
Steroids							1.4	1.1–1.7	0.0002
No	78	84.8	9	52.9	69	92.0			
Yes	14	15.2	8	47.1	6	8.0			

The final multivariate models identified MRI as being significantly more frequently abnormal in patients with sequelae (**Table 6**), that there were more severe cases in adults than children, and that fever, neck stiffness, convulsions and elevated CSF pressure were significantly more frequent in severe cases (**Table 7**).

**Table 7:** Final model of New Caledonian cases of EM according to severity, n = 92.

Variables	Total		Severe		Not severe		Adjusted OR	IC	P value
	N = 92	%	N = 29	%	N = 63	%			
Age							1.01	1.004–1.01	0.001
Mean (SD)	25		34.2(22.7)		20.7 (18)				
Median (IQR)	22.5		36 (27)		15 (30.5)				
Fever							1.31	1.08–1.6	0.007
No	66	71.7	18	62.1	48	76.2			
Yes	26	28.3	11	37.9	15	23.8			
Stiff neck							0.8	0.7–1.006	0.06
No	70	76.1	27	93.1	43	68.3			
Yes	22	23.9	2	6.9	20	6.9			
Convulsion							1.48	1.1–1.9	0.006
No	82	89.1	23	79.3	59	93.7			
Yes	10	10.9	6	20.7	4	6.3			
CSF glucose (g)							1.09	0.99–1.2	0.08
Mean (SD)	2.73		2.9(1.04)		2.63 (0.72)				
Median (IQR)	2.66		2.8 (1.15)		2.6 (0.92)				
Elevated CSF pressure							1.4	1.06–1.84	0.01
No	83	90.2	24	82.8	59	93.7			
Yes	9	9.8	5	17.2	4	6.3			

A subgroup analysis of the 17 patients with confirmed diagnosis of angiostrongyliasis by serology or PCR showed similar characteristics for most of the variables in the univariate analysis.

However, although no statistical difference could be shown between the confirmed cases of neuroangiostrongyliasis and the rest of EM cases we noted a different periodicity as a peak of cases appeared in December (6 cases), the mean duration of stay at the hospital was longer (16.7 vs 12.34, p = 0.38), and a higher proportion of patients were admitted to the ICU (17.6% vs 5.2%, p = 0.18). Steroids were given in a higher proportion of cases (29.4% vs 11.6%, p = 0.15) and CT Scan were done more frequently (70 vs 41% of the cases of patients with EM, p = 0.16). The proportion of severe cases was not higher in this subgroup (29% vs 31%, p = 0.86).

The multivariate analysis showed that increasing age (p = 0.001) and low CSF glucose level (p = 0.006) were associated with severe forms and that radiculitis (p = 0.007) and treatment with steroids (p = 0.02) were associated with death or sequelae (see S1 File).

## Discussion

### Diagnosis and role of angiostrongyliasis in EM

A definitive diagnosis of meningitis due to *A*. *cantonensis* is made by the discovery of the parasite in CSF which is very rare. Serological tests have been available in endemic areas since the 1960s and improved in the 1990s to ensure better specificity with fewer cross reactions. Detection of antibodies produced in response to the infection (immunodiagnosis) can be performed on serum or CSF using enzyme-linked immunosorbent assay (ELISA) or western blot (WB) techniques. However, these methods are not standardized and their diagnostic performance may vary depending on the purity of the native antigenic preparation used [36] which could explain some false negative results. Despite the lack of confirmed neuroangiostrongyliasis diagnosis in the majority of our patients (75 cases), 24 of our cases of EM with CSF eosinophils higher than 40% but without biological confirmation could also be due to *A*. *cantonensis*. In fact, according to a study carried out in Thailand in 2012, a CSF eosinophil count of ≥40% of white blood cells is significantly associated with positive serology for *A*. *cantonensis* [37]. Serology is also a very useful tool to study the seroprevalence of the disease [38].

PCR testing of CSF and serum developed during the 2010s, is fast and significantly enhances the diagnosis [36, 39] when combined with compatible clinical symptoms (headache, meningitis, cranial nerve involvement), laboratory findings (including spinal or blood eosinophilia, positive serology tests), and epidemiological information (known infection of rodents and snails by *A*. *cantonensis* in the region) [37]. In various studies of EM, attribution to *A*. *cantonensis* could be confirmed by serology or PCR for *A*. *cantonensis* in 31 to 65% of the cases [12, 14, 35]. In our study, it was confirmed in 49% of the patients tested by PCR or serology, confirming the major contribution of this parasite to EM in New Caledonia.

Out of 21 PCR tests, 10 were negative: 5 because of alternative diagnosis (toxocariasis in 4 cases, *Streptococcus* A meningitis in 1 case) and 5 perhaps because of bad timing between the onset of symptoms and completion of the test or incomplete exploration of alternative diagnoses.

Alternative diagnosis can often be excluded because of different clinical and/or epidemiological presentation.

Our 17 confirmed cases of neuroangiostrongyliasis showed very similar characteristics to those of EM in patients in whom it was not confirmed to be due to *A*. *cantonensis*. The fact that a higher proportion of patients underwent a CT scan, received steroids and were admitted at the ICU in recent years may have been due to a better knowledge of the disease with rapid appropriate care as the proportion of severe cases was not higher.

### Reservoir of *A*. *cantonensis*

The parasite’s definitive natural hosts are rodents especially rats. The intermediate hosts are snails and slugs, with paratenic hosts including planarians, freshwater shrimp, crabs, and reptiles also contributing to human infections [40]. Risk factors for neuroangiostrongyliasis are ingestion of or contact (mucosa, open wound) with contaminated snails, slugs, water, fruit and vegetables [4, 40], which play a role as intermediate and paratenic hosts. In the 1970s, a study of reservoirs of *A*. *cantonensis* in New Caledonia [27] showed that 30 to 100% of rats were infected depending on species. They therefore play a role as definite hosts. Gastropods (snails, slugs) also seemed to be frequently infected but the most probable route of human infection was thought to be accidental ingestion of infected planarians or slugs on raw vegetables. At that time, the giant African land snail, *Lissachatina fulica* had not yet invaded New Caledonia, so it had not been studied. Currently *L*. *fulica* is responsible for significant damage in vegetable fields and trees and contact with this snail is very frequent among farmers who destroy them by crushing them or collecting them to feed to pigs. Infection could be caused by contact of *L*. *fulica* with the mucosa of the mouth, nose and eyes or more rarely by infective larvae entering wounds caused by crushing of the snail shells.

### Seasonality of incidence of EM cases

There were two peaks of incidence among our EM cases: one in February during the hot and rainy season and one between May and October during the cool dry season unlike in French Polynesia [14]. However, in the study of our 17 confirmed cases, although 6 cases appeared in December, this was not shown to constitute a statistically significant peak. Epelboin et al [35] evoked a link between periodicity of the cases in Mayotte and the route of transmission. But in our situation it would be very hazardous to make any conclusion related to the production of green vegetables in the cool season in New Caledonia favoring the development of slugs and planarians, or the aestivation of *Lissachatina fulica* during the cool season (Fig 1) [41].

### Geography/demography of EM cases

There is a higher incidence of EM cases in the northern humid regions and on the Loyalty Islands but a higher number of cases in the southern province. The inequalities in medical demography could explain this distribution. The density of physicians, in particular of specialists, is higher in the southern province of New Caledonia around Noumea, where the Territorial Hospital of New Caledonia is located [42]. Cases could be underdiagnosed because of difficult access to the health care system in the north and the islands.

Both sexes are affected by EM with a predominance of male cases. In New Caledonia, some activities with probable increased risk of infection are primarily performed by men. Fishing in rivers (with risk of deliberate ingestion of raw freshwater shrimp) is essentially done by men [43]. Farming is usually undertaken by both sexes, with men doing the heavy work like plowing (with risk of exposure to crushed *Lissachatina fulica*) while women maintain the fields (cleaning, pruning, harvesting) [44].

The major incidence among small children between 0 and 4 years old, especially of Melanesian origin may be due to the demographics of the island (39% Melanesian people living predominantly in the Northern Province, 29% Europeans living predominantly in the Southern Province) but could also be explained by different customs and habits [45]. Most of the Melanesians people have their own vegetable gardens and eat many raw vegetables which may be contaminated by planars. Many Melanasians also live close to rivers where they eat crabs or sweet water shrimps which maybe intermediate hosts. *Lissachatina fulica* is responsible for huge damages in the vegetable fields or on trees (for instance papaya) which they climb to pick the fruit and eat it. Contacts with this snail are very frequent in farmers who destroy them by crushing them or collect them to feed the pigs. In contrast, Europeans living in New Caledonia consume more food purchased at the supermarket.

Some studies suggested that the proportion of severe cases was higher in babies and infants [35, 46] but we did not come to the same conclusion in our study.

### Symptoms of EM due to *A*. *cantonensis*

Classically the incubation of EM due to *A*. *cantonensis* lasts for one to two weeks after ingestion, with extremes from 1 to 36 days, and the onset of symptoms can be abrupt or insidious [47]. In our study the mean duration of symptoms before recovery of our confirmed cases was 14.1 days. The main clinical features of EM due to *A*. *cantonensis* usually include nonspecific clinical signs like headache, neck stiffness, and nausea [48], which are more evident in severe cases in our study. They can be less apparent especially in mild cases, so that the disease is probably underdiagnosed [49]. The proportion of cases without sequelae varies greatly from 40 to 98% in the literature (80% in our study), highlighting the need for more epidemiological data about this disease.

### Biology of EM due to *A*. *cantonensis*

In the literature, CSF analysis of patients with EM due to *A*. *cantonensis* usually shows clear, cloudy or puriform hyperdense CSF [47]. Pressure can be above 200 mm of water [47, 50]. Other CSF findings include hypercytosis between 150 and 2000 elements/mm^3^, eosinophilia with upper levels of 10% disappearing around the 12th day, CSF protein rarely above 1 g/L, low CSF glucose [35, 37] and CSF chloride often normal [51]. Parasitological examination of stool is rarely positive as expected based on the life cycle of the parasite [52]. There is often a non-specific intrathecal immunoglobulin synthesis [1]. Blood biology shows hyperleukocytosis: above 10,000 elements/mm^3^ in 75% of cases, explained by hypereosinophilia between 15 and 20% [1, 47]. There is usually no correlation between blood and spinal eosinophilia [12]. In severe forms hyponatraemia caused by inappropriate secretion of antidiuretic hormone (ADH) can be seen [53]. Our study confirmed most of these commonly seen patterns.

### Imaging and other paraclinical features of EM due to *A*. *cantonensis*

Pathological lesions are mainly observed in the CNS and are secondary to the passage of larvae from the blood to the nervous tissue. Other exams can be useful such as brain CT scans (slight ventricular dilation or nonspecific hypodensities), MRI (micronodular enhancements), myelo CT scans (medullary defect), ophthalmological exams, EEGs (non-specific disturbances), EMGs (signs of multiple roots involvement or staging of the anterior horn), negative screening for stool ova and negative blood serology for other parasites [54–56].

In our cases, the CT scans were non-specific showing mostly edema, while the MRI showed diffuse hyperintensities in T1 and 2. The ophthalmological exam was not contributive showing non-specific edema in some cases. The EEG was disturbed only in severe forms and the EMG was mostly normal. The CT scan rarely showed edema of the posterior fossa or ventricular dilatation.

### Treatment of EM due to *A*. *cantonensis*

There is currently no consensus on treatment of angiostrongyliasis though we are moving towards it [57]. Administering albendazole for 14 days was studied in only one randomized and placebo-controlled trial involving 71 subjects and cleared headaches more quickly [58]. Another study showed that the combination of prednisolone plus albendazole was not superior to prednisolone alone. Many authors have suggested that albendazole increases the risk of symptoms and seizures caused by a hyperallergic response to the death of the parasites [59]. For this reason, steroids are commonly used to avoid inflammation resulting from the death of the parasites, to relieve edema and focal deficit, or in case of persistent symptoms. Iterative therapeutic lumbar punctures are also effective to reduce symptoms by decreasing intracranial hypertension [60]. Elevated CSF pressure is multifactorial, partly because of inflammation caused by the natural death of parasites, worsened by antiparasitic treatments and by inappropriate secretion of anti-diuretic hormone [60]. Resuscitation may be needed in severe cases.

In our study, the patients who received steroids had more frequent sequelae probably because those eligible for steroid treatment were already more severe.

If PCR for *A*. *cantonensis* were performed early the alternative diagnoses could be excluded thus avoiding useless and potentially damaging empirical antibiotics.

Prevention consists of controlling hosts, especially rats and terrestrial molluscs. Rats can be controlled with baited traps. Reducing mollusc populations or preventing their access to vegetable plots can be achieved by creating bare strips of land around cultivated areas, employing diverse molluscicides, collecting and disposing of the infested animals, importing ducks in infected areas to kill the infected hosts, and using phytosanitary control of imports. Populations of both rats and snails can be reduced by maintaining a clean, tidy plant growing area that provides limited refuges for these animals. Above all, sanitary education is primary and must include avoiding the consumption of snails, shrimp and crabs that are raw or not previously frozen and cleaning vegetables, especially leafy greens, before consumption. But those public health measures can only be justified once the reservoirs are clearly identified.

### Limitations of our study

Although our study is one of the rare epidemiological descriptive studies [61] of EM cases due to *A*. *cantonensis* in the region, it nevertheless had some limitations. First, case selection was biased because we only studied cases admitted to the Territorial Hospital thus probably excluding many mild cases only seen in peripheral health centers or not at all. Because of the relative rarity of the disease we decided to conduct a retrospective study and there may be some information biases due to variable completeness of data in the medical files. We attempted to overcome confounding bias by performing a multivariate logistic regression model allowing an adjustment to the other explanatory variables.

### Perspectives

As the treatment of EM due to *A*. *cantonensis* is essentially symptomatic, identifying reservoirs and educating the population and health professionals are the most effective measures to prevent the disease. Facing diverse clinical symptoms, the severe forms and the frequent sequelae, we could consider extending the indications for lumbar punctures in New Caledonia in order to better diagnose the disease.

The diagnosis algorithm for EM should be known by health professionals in New Caledonia and in the case of negative PCR and serology for *A*. *cantonensis*, some alternative diagnoses should systematically be considered (toxocariasis, cysticercosis, gnathostomiasis). MRI lesions are more frequently observed in severe forms. Imaging should be performed in case of encephalitis or radiculitis to identify potential complications.

## Conclusion

In New Caledonia, about 5.8 cases of eosinophilic meningitis occur each year, which represents an incidence of about 2.1 /100,000 inhabitants/year. The presentation is diverse, sometimes severe and sequelae are frequent. The disease is probably underdiagnosed as the most frequent symptoms are nonspecific. There appears to be a higher incidence in young children. Eosinophilic meningitis has often been attributed to the parasite *A*. *cantonensis* without biological confirmation as PCR has only been available since 2014 and serology is not often performed. Many patients are treated with albendazole despite the lack of evidence of any medical benefit. The incidence reported justifies additional investigation into the potential reservoirs and education of physicians and the public in of New Caledonia (in accordance with the proposals of the 2012 workshop in Hawaii) [3]. We could then better target methods of prevention such as washing raw vegetables and cooking seafood.

## Supporting information

S1 File Features of New Caledonian cases of angiostrongyliasis from 2004 to 2019 according to severity and death/sequelae, n = 17. (DOCX)
