# Supplementary material for: Eosinophilic meningitis in New Caledonia: The role of Angiostrongylus cantonensis?
Source: PLoS One. 2021 Aug 12;16(8):e0254964. doi: 10.1371/journal.pone.0254964 (PMC8360557; doi:10.1371/journal.pone.0254964)
Supplement: S1 File — (DOCX) [file pone.0254964.s001.docx]

**Tables**

**Table 1: Socio-demographical features of New Caledonian cases of angiostrongyliasis from 2004 to 2019 according to severity, n=17**

| **Variables** | **Total** | | | | | | | **Severe** | | | | | **Non severe** | | | | | | **Crude OR** | | **95% CI** | | **P value** |
| --- | --- | --- | --- | --- | --- | --- | --- | --- | --- | --- | --- | --- | --- | --- | --- | --- | --- | --- | --- | --- | --- | --- | --- |
|  | **N=17** | | | **%** | | | | **N=5** | | **%** | | | **N=12** | | | | | **%** |  |  |  |  |  |
| Age | | |  | | |  |  | | | | |  | | | |  | | | 1.08 | | 1.01-1.15 | | 0.03 |
| Mean (SD) | 26.2 | | |  | | | 52.4 (15) | | | | | | | | 15.33 (22) | | | |  | |  |  |  |
| Median (IQR) | 12 | | |  | | | 54 (18) | | | | | | | | 5 (15.7) | | | |  | |  |  |  |
| Age class (years old) | | | |  | | | |  | |  | | |  | | | | |  |  | |  | |  |
| 0-4 | 3 | | | 17.6 | | | | 0 | | 0.0 | | | 3 | | | | | 25.0 |  | |  | |  |
| 5-18 | 6 | | | 35.3 | | | | 0 | | 0.0 | | | 6 | | | | | 50.0 |  | |  | |  |
| 19-25 | 0 | | | 0 | | | 0 | | | 0.0 | | | | | 0 | | | 0.0 |  | |  | |  |
| >25 | 8 | | | 47.1 | | | 5 | | | 100.0 | | | | | 3 | | | 25.0 |  | |  | |  |
| Sex masculine | |  | | |  | | | |  | |  | | |  | | |  | | | 0.75 | 0.09-6.5 | | 0.79 |
| No | | 6 | | | 35.3 | | | | 2 | | 40.0 | | | 4 | | | 33.3 | | |  |  | |  |
| Yes | | 11 | | | 64.7 | | | | 3 | | 60.0 | | | 8 | | | 66.7 | | |  |  | |  |
| Melanesian | |  | | |  | | | |  | |  | | |  | | |  | | |  |  | |  |
| No | | 0 | | | 0 | | | | 0 | | 0.0 | | | 0 | | | 0.0 | | |  |  | |  |
| Yes | | 17 | | | 100 | | | | 5 | | 100.0 | | | 12 | | | 100.0 | | |  |  | |  |
| Rural | |  | | |  | | | |  | |  | | |  | | |  | | |  |  | |  |
| No | | 2 | | | 11.8 | | | | 0 | | 0.0 | | | 2 | | | 16.7 | | |  |  | |  |
| Yes | | 15 | | | 88.2 | | | | 5 | | 100.0 | | | 10 | | | 83.3 | | |  |  | |  |
| Province | |  | | |  | | | |  | |  | | |  | | |  | | |  |  | |  |
| Islands | | 2 | | | 11.8 | | | | 0 | | 0.0 | | | 2 | | | 16.7 | | |  |  | |  |
| North | | 6 | | | 35.3 | | | | 2 | | 40.0 | | | 4 | | | 33.3 | | |  |  | |  |
| South | | 7 | | | 41.2 | | | | 1 | | 20.0 | | | 6 | | | 50.0 | | |  |  | |  |
| Others (Vanuatu) | | 2 | | | 11.8 | | | | 2 | | 40.0 | | | 0 | | | 0.0 | | |  |  | |  |
| Seasonality | |  | | |  | | | |  | |  | | |  | | |  | | | 0.67 | 0.08-5.54 | | 0.7 |
| May October | | 8 | | | 47.1 | | | | 3 | | 60.0 | | | 6 | | | 50.0 | | |  |  | |  |
| November April | | 9 | | | 52.9 | | | | 2 | | 40.0 | | | 6 | | | 50.0 | | |  |  | |  |
|  |  | | |  | | |  | | |  | | | | |  | | |  |  | |  | |  |

**Table 2: Clinical features of New Caledonian cases of angiostrongyliasis from 2004 to 2019 according to severity, n=17**

| **Variables** | **Total** | | **Severe** | | | **Not severe** | | | **Crude OR** | **95% CI** | **P value** |
| --- | --- | --- | --- | --- | --- | --- | --- | --- | --- | --- | --- |
|  | **N=17** | **%** | **N=5** | | **%** | **N=12** | | **%** |  |  |  |
| Duration of symptoms (d) | |  |  | | |  | | | 2.23 | 8.4-19.1 | 0.001 |
| Mean (SD) | 14.1 |  | 19.7(12) | |  | 12.4(7.7) | | |  |  |  |
| Median (IQR) | 11.0 |  | 21(12) | |  | 10.5(7.25) | | |  |  |  |
| Duration of hospitalization (d) | | |  | |  |  | | | 1.31 | 0.87-1.96 | 0.2 |
| Mean (SD) | 16.65 |  | 42.8 (61.1) | | | 5.75 (3.9) | | |  |  |  |
| Median (IQR) | 7 |  | 11(28) | | | 5(7) | | |  |  |  |
| ICU |  |  |  | | |  | | | 1.2 | 0.01-30 | 1 |
| No | 14 | 82.4 | 4 | 80.0 | | 10 | 83.3 | |  |  |  |
| Yes | 3 | 17.6 | 1 | 20.0 | | 2 | 16.7 | |  |  |  |
| Headache |  |  |  | |  |  | |  | 0.22 | 0.02-2.04 | 0.18 |
| No | 6 | 35.3 | 3 | | 60.0 | 3 | | 25.0 |  |  |  |
| Yes | 11 | 64.7 | 2 | | 40.0 | 9 | | 75.0 |  |  |  |
| Fever |  |  |  | |  |  | |  | 0.67 | 0.08-5.54 | 0.7 |
| No | 9 | 52.9 | 3 | | 60.0 | 6 | | 50.0 |  |  |  |
| Yes | 8 | 47.1 | 2 | | 40.0 | 6 | | 50.0 |  |  |  |
| Crying |  |  |  | |  |  | |  |  |  |  |
| No | 2 | 11.8 | 0 | | 0.0 | 2 | | 16.7 |  |  |  |
| Yes | 15 | 88.2 | 5 | | 100.0 | 10 | | 83.3 |  |  |  |
| Deterioration of general condition |  |  |  | |  |  | |  | 0.08 | 0.01-1.0 | 0.03 |
| No | 7 | 41.2 | 4 | | 80.0 | 3 | | 25.0 |  |  |  |
| Yes | 10 | 58.8 | 1 | | 20.0 | 9 | | 75.0 |  |  |  |
| Grey complexion |  |  |  | |  |  | |  |  |  |  |
| No | 15 | 88.2 | 5 | | 100.0 | 10 | | 83.3 |  |  |  |
| Yes | 2 | 11.8 | 0 | | 0.0 | 2 | | 16.7 |  |  |  |
| Coma |  |  |  | |  |  | |  |  |  |  |
| No | 16 | 94.1 | 4 | | 80.0 | 12 | | 100.0 |  |  |  |
| Yes | 1 | 5.9 | 1 | | 20.0 | 0 | | 0.0 |  |  |  |
| Drowsiness |  |  |  | |  |  | |  |  |  |  |
| No | 13 | 76.5 | 5 | | 100.0 | 8 | | 66.7 |  |  |  |
| Yes | 4 | 23.5 | 0 | | 0.0 | 4 | | 33.3 |  |  |  |
| Vomiting |  |  |  | |  |  | |  | 0.08 | 0.01-1.07 | 0.05 |
| No | 7 | 41.2 | 4 | | 80.0 | 3 | | 25.0 |  |  |  |
| Yes | 10 | 58.8 | 1 | | 20.0 | 9 | | 75.0 |  |  |  |
| Stiff neck |  |  |  | |  |  | |  |  |  |  |
| No | 13 | 76.5 | 5 | | 100.0 | 8 | | 66.7 |  |  |  |
| Yes | 4 | 23.5 | 0 | | 0.0 | 4 | | 33.3 |  |  |  |
| Convulsion |  |  |  | |  |  | |  |  |  |  |
| No | 16 | 94.1 | 5 | | 100.0 | 11 | | 91.7 |  |  |  |
| Yes | 1 | 5.9 | 0 | | 0.0 | 1 | | 8.3 |  |  |  |

**Table 3: Biological features of the New Caledonian cases of angiostrongyliasis from 2004 to 2019 according to severity, n=17**

| **Variables** | **Total** | | **Severe** | | | | **Not severe** | | **Crude OR** | **95% CI** | | **P value** |
| --- | --- | --- | --- | --- | --- | --- | --- | --- | --- | --- | --- | --- |
|  | **N=17** | **%** | **N=5** | | | **%** | **N=12** | **%** |  |  |  |  |
| % CSF eosinophils |  |  | |  | |  |  |  | 1.03 | 0.97-1.09 | 0.32 | |
| Mean (SD) | 36.47 |  | | 43.4 (16.9) | | | 33.6 (19.5) | |  |  |  | |
| Median (IQR) | 31 |  | | 45 (26) | | | 30.5(20.3) | |  |  |  | |
| CSF eosinophils >30% | |  | |  | | |  | | 1.65 | 0.37-6.8 | 0.5 | |
| No | 80 | 87.0 | | 24 | 82.8 | | 56 | 88.9 |  |  |  | |
| Yes | 12 | 13.0 | | 5 | 17.2 | | 7 | 11.1 |  |  |  | |
| CSF GB (/mm3) |  |  | |  | |  |  |  | 1 | 0.99-1 | 0.22 | |
| Mean (SD) | 502 |  | | 333 (144) | | | 572(408) | |  |  |  | |
| Median (IQR) | 500 |  | | 381 (220) | | | 577(491) | |  |  |  | |
| CSF proteins (g/L) |  |  | |  | |  |  |  | 7.8 | 0.63-98 | 0.1 | |
| Mean (SD) | 1.33 |  | | 2.7 (2.2) | | | 0.7(0.44) | |  |  |  | |
| Median (IQR) | 0.8 |  | | 2.17 (3) | | | 0.72(0.55) | |  |  |  | |
| CSF proteins > 1 g |  |  | |  | |  |  |  | 3 | 0.35-25.8 | 0.3 | |
| No | 10 | 58.8 | | 2 | | 40.0 | 8 | 66.7 |  |  |  | |
| Yes | 7 | 41.2 | | 3 | | 60.0 | 4 | 33.3 |  |  |  | |
| CSF glucose (g) |  |  | |  | |  |  |  | 2.7 | 0.7-9.5 | 0.12 | |
| Mean (SD) | 2.72 |  | | 3.6 (1.9) | | | 2.3 (0.74) | |  |  |  | |
| Median (IQR) | 2.66 |  | | 3.6 (1.2) | | | 2.6 (0.7) | |  |  |  | |
| Blood eosinophils (/mm3) | |  | |  | |  |  |  | 1 | 0.99-1 | 0.2 | |
| Mean (SD) | 1848 |  | | 1074 (924) | | | 2170 (1610) | |  |  |  | |
| Median (IQR) | 1220 |  | | 940 (320) | | | 2095 (2580) | |  |  |  | |
| % blood eosinophils | | | |  | |  |  |  | 0.95 | 0.83-1.08 | 0.4 | |
| Mean (SD) | 13.8 |  | | 11.2 (7.1) | | | 14.9 (9.4) | |  |  |  | |
| Median (IQR) | 16.0 |  | | 10.7 (9.1) | | | 16.0 (10.6) | |  |  |  | |
| Elevated CSF pressure | |  | |  | |  |  |  |  |  |  | |
| No | 15 | 88.2 | | 5 | 100.0 | | 10 | 83.3 |  |  |  | |
| Yes | 2 | 11.8 | | 0 | 0.0 | | 2 | 16.7 |  |  |  | |
| Positive serology |  |  | |  | |  |  |  | 0.22 | 0.02-2.04 | 0.18 | |
| No | 11 | 64.7 | | 2 | 40.0 | | 9 | 75.0 |  |  |  | |
| Yes | 6 | 35.3 | | 3 | 60.0 | | 3 | 25.0 |  |  |  | |
| Positive CSF serology | |  | |  | |  |  |  | 0.06 | 0-0.92 | 0.04 | |
| No | 13 | 76.5 | | 2 | | 40.0 | 11 | 91.7 |  |  |  | |
| Yes | 4 | 23.5 | | 3 | | 60.0 | 1 | 8.3 |  |  |  | |
| Positive CSF PCR |  |  | |  | |  |  |  | 0.22 | 0.02-2.04 | 0.18 | |
| No | 6 | 35.3 | | 3 | | 60.0 | 3 | 25.0 |  |  |  | |
| Yes | 11 | 64.7 | | 2 | | 40.0 | 9 | 75.0 |  |  |  | |

**Table 4: Radiological features of New Caledonian cases of angiostrongyliasis from 2004 to 2019 according to severity, n=17**

| **Variables** | **Total** | | **Severe** | | **Not severe** | | **Crude OR** | **95% CI** | **P value** |
| --- | --- | --- | --- | --- | --- | --- | --- | --- | --- |
|  | **N=17** | **%** | **N=5** | **%** | **N=12** | **%** |  |  |  |
| Abnormal CT scan |  |  |  |  |  |  | 2 | 0.22-18.3 | 0.5 |
| No | 12 | 70.6 | 3 | 60.0 | 9 | 75.0 |  |  |  |
| Yes | 5 | 29.4 | 2 | 40.0 | 3 | 25.0 |  |  |  |
| Abnormal MRI |  |  |  |  |  |  |  |  |  |
| No | 15 | 88.2 | 3 | 60.0 | 12 | 100.0 |  |  |  |
| Yes | 2 | 11.8 | 2 | 40.0 | 0 | 0.0 |  |  |  |
| Abnormal EEG |  |  |  |  |  |  |  |  |  |
| No | 14 | 82.4 | 5 | 100.0 | 9 | 75.0 |  |  |  |
| Yes | 3 | 17.6 | 0 | 0.0 | 3 | 25.0 |  |  |  |
| Abnormal fundoscopic examination |  |  |  |  |  |  |  |  |  |
| No | 17 | 100.0 | 5 | 100.0 | 12 | 100.0 |  |  |  |
| Yes | 0 | 0.0 | 0 | 0.0 | 0 | 0.0 |  |  |  |

**Table 5: Treatment of New Caledonian cases of angiostrongyliasis according to severity, n=17**

| **Variables** | **Total** | | **Severe** | | | **Not severe** | | **Crude OR** | **95% CI** | | **P value** |
| --- | --- | --- | --- | --- | --- | --- | --- | --- | --- | --- | --- |
|  | **N=17** | **%** | **N=5** | | **%** | **N=12** | **%** |  |  |  |  |
| Albendazole |  |  | |  |  |  |  | 2 | 0.22-18.3 | 0.5 | |
| No | 12 | 70.6 | | 3 | 60.0 | 9 | 75.0 |  |  |  | |
| Yes | 5 | 29.4 | | 2 | 40.0 | 3 | 25.0 |  |  |  | |
| Duration of albendazole (d) | |  | |  |  |  |  |  |  | 0.39 | |
| Mean | 3 |  | | 3 (0) |  | 3 (0) |  |  |  |  | |
| Median | 3 |  | | 3 (0) |  | 3 (0) |  |  |  |  | |
| Ivermectin |  |  | |  |  |  |  |  |  |  | |
| No | 16 | 94.1 | | 4 | 80.0 | 12 | 100.0 |  |  |  | |
| Yes | 1 | 5.9 | | 1 | 20.0 | 0 | 0.0 |  |  |  | |
| Duration of ivermectin (d) | |  | |  |  |  |  |  |  | 0.7 | |
| Mean | 2 |  | | 2(0) |  | 2 (0) |  |  |  |  | |
| Median | 2 |  | | 2(0) |  | 2 (0) |  |  |  |  | |
| Steroids |  |  | |  |  |  |  | 7.5 | 0.7-78.3 | 0.09 | |
| No | 12 | 70.6 | | 2 | 40.0 | 10 | 83.3 |  |  |  | |
| Yes | 5 | 29.4 | | 3 | 60.0 | 2 | 16.7 |  |  |  | |
| Duration of steroids (d) |  |  | |  |  |  |  | 1.8 | 0.25-33 | 0.12 | |
| Mean | 33.5 |  | | 65 (43) |  | 2 (1.7) |  |  |  |  | |
| Median | 9 |  | | 90 (38) |  | 3 (1.5) |  |  |  |  | |
| Subtractive lumbar puncture |  |  | |  |  |  |  |  |  |  | |
| No | 16 | 94.1 | | 4 | 100.0 | 12 | 92.3 |  |  |  | |
| Yes | 1 | 5.9 | | 0 | 0.0 | 1 | 7.7 |  |  |  | |
| Antibiotics |  |  | |  |  |  |  |  |  |  | |
| No | 15 | 88.2 | | 5 | 100.0 | 10 | 83.7 |  |  |  | |
| Yes | 2 | 11.8 | | 0 | 0.0 | 2 | 16.7 |  |  |  | |

**Table 6: Final model of New Caledonian cases of angiostrongyliasis according to severity, n=17**

| **Variables** | | | **Total** | | | | | | | | | **Severe** | | | | | | **Not severe** | | | | | | | **Adjusted OR** | | | | **IC** | | | | **P value** | | |  |
| --- | --- | --- | --- | --- | --- | --- | --- | --- | --- | --- | --- | --- | --- | --- | --- | --- | --- | --- | --- | --- | --- | --- | --- | --- | --- | --- | --- | --- | --- | --- | --- | --- | --- | --- | --- | --- |
|  | | | **N=17** | | | | **%** | | | | | **N=5** | | **%** | | | | **N=12** | | | | | **%** | |  |  |  |  |  |  |  |  |  |  |  |  |
| Age | | | |  | | | |  | | |  | | | | |  | | | | | |  | | | | 1.01 | | | 1.004-1.01 | | | | | | 0.001 | |
| Mean (SD) | | 25 | | | |  | | | | | 34.2(22.7) | | | | | | | | 20.7 (18) | | | | | | |  | | |  | | |  | | |  | |
| Median (IQR) | | 22.5 | | | |  | | | | | 36 (27) | | | | | | | | 15 (30.5) | | | | | | |  | | |  | | |  | | |  | |
| Headache |  | | | |  | | | | |  | | | | |  | | | | |  | | | |  | | | | 0.8 | | | 0.6-1.01 | | | 0.1 | | |
| No | 6 | | | | 35.3 | | | | | 3 | | | | | 60.0 | | | | | 3 | | | | 25.0 | | | |  | | |  | | |  | | |
| Yes | 11 | | | | 64.7 | | | | | 2 | | | | | 40.0 | | | | | 9 | | | | 75.0 | | | |  | | |  | | |  | | |
| CSF glucose (g) |  | | | |  | | | | |  | | | | |  | | | | |  | | | |  | | | | 1.2 | | | 1.1-1.42 | | | 0.006 | | |
| Mean (SD) | 2.72 | | | |  | | | | | 3.6(1.9) | | | | | 2.3(0.7) | | | | | |  | | |  | | | |  | | |  | | |  | | |
| Median (IQR) | 2.66 | | | |  | | | | | 3.6(1.2) | | | | | 2.6(0.7) | | | | | |  | | |  | | | |  | | |  | | |  | | |
| CSF proteins > 1 g |  | | | |  | | | | |  | | | | |  | | | | | |  | | |  | | | | 0.97 | | | 0.86-1.07 | | | 0.5 | | |
| No | 10 | | | | 58.8 | | | | | 2 | | | | | 40.0 | | | | | | 8 | | | 66.7 | | | |  | | |  | | |  | | |
| Yes | 7 | | | | 41.2 | | | | | 3 | | | | | 60.0 | | | | | | 4 | | | 33.3 | | | |  | | |  | | |  | | |
| Blood eosinophils (/mm3) |  | | | |  | | | |  | | | | | |  | | | | | |  | | |  | | | | 1 | | | 0.99-1 | | | 0.48 | | |
| Mean (SD) | 1848 | | | |  | | | | 1074 (924) | | | | | | 2170 (1610) | | | | | | | | |  | | | |  | | |  |  |  |  |  |  |
| Median (IQR) | 1220 | | | |  | | | | 940 (320) | | | | | | 2095 (2580) | | | | | | | | |  | | | |  | | |  |  |  |  |  |  |
| Positive serology | |  | | | |  | | | | | |  |  | | | |  | | | | | |  | | | | 0.88 | | | 0.57-1.36 | | | 0.59 | | |  |
| No | | 11 | | | | 64.7 | | | | | | 2 | 40.0 | | | | 9 | | | | | | 75.0 | | | |  | | |  | | |  | | |  |
| Yes | | 6 | | | | 35.3 | | | | | | 3 | 60.0 | | | | 3 | | | | | | 25.0 | | | |  | | |  | | |  | | |  |
| Positive CSF serology | |  | | | |  | | | | | |  |  | | | |  | | | | | |  | | | | 0.64 | | | 0.4-0.98 | | | 0.07 | | |  |
| No | | 13 | | | | 76.5 | | | | | | 2 | 40.0 | | | | 11 | | | | | | 91.7 | | | |  | | |  | | |  | | |  |
| Yes | | 4 | | | | 23.5 | | | | | | 3 | 60.0 | | | | 1 | | | | | | 8.3 | | | |  | | |  | | |  | | |  |

**Table 7: Socio-demographical features of New Caledonian cases of angiostrongyliasis from 2004 to 2019 according to death/sequelae, n=17**

| **Variables** | **Total** | | | | | | | **Death** | | | | | **Without sequelae** | | | | | | **Crude OR** | | **95% CI** | | **P value** |
| --- | --- | --- | --- | --- | --- | --- | --- | --- | --- | --- | --- | --- | --- | --- | --- | --- | --- | --- | --- | --- | --- | --- | --- |
|  | **N=17** | | | **%** | | | | **N=4** | | **%** | | | **N=13** | | | | | **%** |  |  |  |  |  |
| Age | | |  | | |  |  | | | | |  | | | |  | | | 1.06 | | 1.1-1.12 | | 0.0023 |
| Mean (SD) | 26.2 | | |  | | | 51 (18) | | | | | | | | 18.5 (24) | | | |  | |  |  |  |
| Median (IQR) | 12 | | |  | | | 48.5 (25) | | | | | | | | 7(32) | | | |  | |  |  |  |
| Age class (years old) | | | |  | | | |  | |  | | |  | | | | |  |  | |  | | 0.024 |
| 0-4 | 3 | | | 17.6 | | | | 0 | | 0.0 | | | 3 | | | | | 23.1 |  | |  | |  |
| 5-18 | 6 | | | 35.3 | | | | 0 | | 0.0 | | | 6 | | | | | 46.2 | 0.33 | | 0.02-6.65 | | 0.47 |
| 19-25 | 0 | | | 0 | | | 0 | | | 0.0 | | | | | 0 | | | 0 | 2.55 | | 0.4-15.4 | | 0.21 |
| >25 | 8 | | | 47.1 | | | 4 | | | 100.0 | | | | | 4 | | | 30.8 | 2.58 | | 0.8-8.26 | | 0.376 |
| Sex masculine | |  | | |  | | | |  | |  | | |  | | |  | | | 0.44 | 0.05-4.37 | | 0.5 |
| No | | 6 | | | 35.3 | | | | 2 | | 50.0 | | | 4 | | | 30.8 | | |  |  | |  |
| Yes | | 11 | | | 64.7 | | | | 2 | | 50.0 | | | 9 | | | 69.2 | | |  |  | |  |
| Melanesian | |  | | |  | | | |  | |  | | |  | | |  | | |  |  | |  |
| No | | 0 | | | 0 | | | | 0 | | 0 | | | 0 | | | 0 | | |  |  | |  |
| Yes | | 17 | | | 100.0 | | | | 4 | | 100.0 | | | 13 | | | 100 | | |  |  | |  |
| Rural | |  | | |  | | | |  | |  | | |  | | |  | | | 4.0 | 0.19-84.2 | | 0.37 |
| No | | 2 | | | 11.8 | | | | 1 | | 25.0 | | | 1 | | | 7.7 | | |  |  | |  |
| Yes | | 15 | | | 88.2 | | | | 3 | | 75.0 | | | 12 | | | 92.3 | | |  |  | |  |
| Province | |  | | |  | | | |  | |  | | |  | | |  | | |  |  | |  |
| Islands | | 2 | | | 11.8 | | | | 0 | | 0.0 | | | 2 | | | 15.4 | | |  |  | |  |
| North | | 6 | | | 35.3 | | | | 2 | | 50.0 | | | 4 | | | 30.8 | | |  |  | |  |
| South | | 7 | | | 41.2 | | | | 2 | | 50.0 | | | 5 | | | 38.5 | | |  |  | |  |
| Others (Vanuatu, Wallis) | | 2 | | | 11.8 | | | | 0 | | 0.0 | | | 2 | | | 15.4 | | |  |  | |  |
| Seasonality | |  | | |  | | | |  | |  | | |  | | |  | | | 1.17 | 0.12-10.99 | | 0.89 |
| May-October | | 8 | | | 47.1 | | | | 2 | | 50.0 | | | 6 | | | 46.2 | | |  |  | |  |
| November-April | | 9 | | | 52.9 | | | | 2 | | 50.0 | | | 7 | | | 53.8 | | |  |  | |  |
|  |  | | |  | | |  | | |  | | | | |  | | |  |  | |  | |  |

**Table 8: Clinical features of New Caledonian cases of angiostrongyliasis from 2004 to 2019 according to death/sequelae, n=17**

| **Variables** | **Total** | | **Death** | | | **Without sequelae** | | | **Crude OR** | **95% CI** | **P value** |
| --- | --- | --- | --- | --- | --- | --- | --- | --- | --- | --- | --- |
|  | **N=17** | **%** | **N=4** | | **%** | **N=13** | | **%** |  |  |  |
| Duration of symptoms (d) | |  |  | | |  | | | 8.4-19 | 1.5-2.9 | 0.0001 |
| Mean (SD) | 14 |  | 31(0) | |  | 11(5.11) | | |  |  |  |
| Median (IQR) | 11 |  | 31(0) | |  | 10(8) | | |  |  |  |
| Duration of hospitalization (d) | | |  | |  |  | | | 0.99 | 0.96-1.03 | 0.96 |
| Mean (SD) | 16.65 |  | 16 (14) | | | 16.8 (40.2) | | |  |  |  |
| Median (IQR) | 7 |  | 10 (9) | | | 5(7) | | |  |  |  |
| ICU |  |  |  | | |  | | |  |  |  |
| No | 14 | 82.4 | 4 | 100.0 | | 10 | 76.9 | |  |  |  |
| Yes | 3 | 17.6 | 0 | 0.0 | | 3 | 23.1 | |  |  |  |
| Headache |  |  |  | |  |  | |  | 0.44 | 0.05-4.37 | 0.49 |
| No | 6 | 35.3 | 2 | | 50.0 | 4 | | 30.8 |  |  |  |
| Yes | 11 | 64.7 | 2 | | 50.0 | 9 | | 69.2 |  |  |  |
| Fever |  |  |  | |  |  | |  | 0.29 | 0.02-3.52 | 0.33 |
| No | 9 | 52.9 | 3 | | 75 | 6 | | 46.3 |  |  |  |
| Yes | 8 | 47.1 | 1 | | 25 | 7 | | 53.8 |  |  |  |
| Crying |  |  |  | |  |  | |  |  |  |  |
| No | 15 | 88.2 | 4 | | 100.0 | 11 | | 84.6 |  |  |  |
| Yes | 2 | 11.8 | 0 | | 0.0 | 2 | | 15.4 |  |  |  |
| Deterioration of general condition |  |  |  | |  |  | |  | 0.6 | 0.07-5.97 | 0.68 |
| No | 7 | 41.2 | 2 | | 50.0 | 5 | | 38.5 |  |  |  |
| Yes | 10 | 58.8 | 2 | | 50.0 | 8 | | 61.5 |  |  |  |
| Grey complexion |  |  |  | |  |  | |  |  |  |  |
| No | 15 | 88.2 | 4 | | 100.0 | 11 | | 84.6 |  |  |  |
| Yes | 2 | 11.8 | 0 | | 0.0 | 2 | | 15.4 |  |  |  |
| Coma |  |  |  | |  |  | |  |  |  |  |
| No | 16 | 94.1 | 4 | | 100.0 | 12 | | 92.3 |  |  |  |
| Yes | 1 | 5.9 | 0 | | 0.0 | 1 | | 7.7 |  |  |  |
| Drowsiness |  |  |  | |  |  | |  | 1.11 | 0.08.-15.0 | 0.94 |
| No | 13 | 76.5 | 3 | | 75.0 | 10 | | 76.9 |  |  |  |
| Yes | 4 | 23.5 | 1 | | 25.0 | 3 | | 23.1 |  |  |  |
| Vomiting |  |  |  | |  |  | |  | 0.15 | 0.01-1.9 | 0.14 |
| No | 7 | 41.2 | 3 | | 75.0 | 4 | | 30.8 |  |  |  |
| Yes | 10 | 58.8 | 1 | | 25.0 | 9 | | 69.2 |  |  |  |
| Stiff neck |  |  |  | |  |  | |  |  |  |  |
| No | 13 | 76.5 | 4 | | 100.0 | 9 | | 69.2 |  |  |  |
| Yes | 4 | 23.5 | 0 | | 0.0 | 4 | | 30.8 |  |  |  |
| Convulsion |  |  |  | |  |  | |  |  |  |  |
| No | 16 | 94.1 | 4 | | 100.0 | 12 | | 92.3 |  |  |  |
| Yes | 1 | 5.9 | 0 | | 0.0 | 1 | | 7.7 |  |  |  |
| Encephalitis |  |  |  | |  |  | |  |  |  |  |
| No | 16 | 94.1 | 4 | | 100.0 | 12 | | 92.3 |  |  |  |
| Yes | 1 | 5.9 | 0 | | 0.0 | 1 | | 7.7 |  |  |  |
| Radiculitis |  |  |  | |  |  | |  | 36 | 1.71-758 | 0.02 |
| No | 13 | 76.5 | 1 | | 25.0 | 12 | | 92.3 |  |  |  |
| Yes | 4 | 23.5 | 3 | | 75.0 | 1 | | 7.7 |  |  |  |
| Cranial nerves |  |  |  | |  |  | |  | 36 | 1.71-758 | 0.02 |
| No | 13 | 76.5 | 1 | | 25.0 | 12 | | 92.3 |  |  |  |
| Yes | 4 | 23.5 | 3 | | 75.0 | 1 | | 7.7 |  |  |  |
|  |  |  |  | |  |  | |  |  |  |  |
|  |  |  |  | |  |  | |  |  |  |  |

**Table 9: Biological features of the New Caledonian cases of angiostrongyliasis from 2004 to 2019 according to death/sequelae, n=17**

| **Variables** | **Total** | | **Death** | | | | **Without sequelae** | | **Crude**  **OR** | **95% CI** | | **P value** |
| --- | --- | --- | --- | --- | --- | --- | --- | --- | --- | --- | --- | --- |
|  | **N=17** | **%** | **N=4** | | | **%** | **N=13** | **%** |  |  |  |  |
| % CSF eosinophils |  |  | |  | |  |  |  | 1.02 | 0.96-1.08 | 0.53 | |
| Mean (SD) | 36.5 |  | | 41.5(11.5) | | | 34.9 (20.7) | |  |  |  | |
| Median (IQR) | 31 |  | | 40 (14) | | | 30(33) | |  |  |  | |
| CSF eosinophils >30% | |  | |  | | |  | |  |  |  | |
| No | 15 | 88.2 | | 4 | 100.0 | | 11 | 84.6 |  |  |  | |
| Yes | 2 | 11.8 | | 0 | 0.0 | | 2 | 15.4 |  |  |  | |
| CSF WBC (/mm3) |  |  | |  | |  |  |  | 0.99 | 0.99-1 | 0.58 | |
| Mean (SD) | 502.2 |  | | 419 (300) | | | 527 (388) | |  |  |  | |
| Median (IQR) | 500 |  | | 350 (386) | | | 510 (400) | |  |  |  | |
| CSF proteins (g/L) |  |  | |  | |  |  |  | 0.89 | 0.38-2.13 | 0.79 | |
| Mean (SD) | 1.32 |  | | 1.16 (0.67) | | | 1.38(1.7) | |  |  |  | |
| Median (IQR) | 0.8 |  | | 0.86 (0.39) | | | 0.76 (0.93) | |  |  |  | |
| CSF proteins > 1 g |  |  | |  | |  |  |  | 0.4 | 0.03-4.8 | 0.46 | |
| No | 10 | 58.8 | | 3 | | 75.0 | 7 | 53.8 |  |  |  | |
| Yes | 7 | 41.2 | | 1 | | 25.0 | 6 | 46.2 |  |  |  | |
| CSF glucose (g) |  |  | |  | |  |  |  | 1.004 | 0.4-2.4 | 0.99 | |
| Mean (SD) | 2.72 |  | | 2.74(1.39) | | | 2.71 (0.72) | |  |  |  | |
| Median (IQR) | 2.66 |  | | 2.85 (1.12) | | | 2.58 (0.9) | |  |  |  | |
| Blood eosinophils (/mm3) | |  | |  | |  |  |  | 0.99 | 0.99-1 | 0.27 | |
| Mean (SD) | 1848 |  | | 1182 (1035) | | | 2052.3 (1598) | |  |  |  | |
| Median (IQR) | 1220 |  | | 1010 (637.5) | | | 1610 (2630) | |  |  |  | |
| % blood eosinophils | | | |  | |  |  |  | 1.00 | 0.8-1.14 | 0.99 | |
| Mean (SD) | 13.81 |  | | 13.8 (8.3) | | | 13.8 (9.2) | |  |  |  | |
| Median (IQR) | 16 |  | | 17.45 (5.1) | | | 12.7 (10.1) | |  |  |  | |
| Elevated CSF pressure | |  | |  | |  |  |  |  |  |  | |
| No | 17 | 100.0 | | 4 | 100.0 | | 13 | 100.0 |  |  |  | |
| Yes | 0 | 0.0 | | 0 | 0.0 | | 0 | 0.0 |  |  |  | |
| Positive serology |  |  | |  | |  |  |  | 0.44 | 0.05-4.37 | 0.49 | |
| No | 11 | 64.7 | | 2 | 50.0 | | 9 | 69.2 |  |  |  | |
| Yes | 6 | 35.3 | | 2 | 50.0 | | 4 | 30.8 |  |  |  | |
| Positive CSF serology | |  | |  | |  |  |  | 0.18 | 0.02-2.15 | 0.18 | |
| No | 13 | 76.5 | | 2 | | 50.0 | 11 | 84.6 |  |  |  | |
| Yes | 4 | 23.5 | | 2 | | 50.0 | 2 | 15.4 |  |  |  | |
| Positive CSF PCR |  |  | |  | |  |  |  | 0.44 | 0.05-4.37 | 0.49 | |
| No | 6 | 35.3 | | 2 | | 50.0 | 4 | 30.8 |  |  |  | |
| Yes | 11 | 64.7 | | 2 | | 50.0 | 9 | 69.2 |  |  |  | |

**Table 10: Radiological features of New Caledonian cases of angiostrongyliasis from 2004 to 2019 according to death/sequelae, n=17**

| **Variables** | **Total** | | **Severe** | | **Not severe** | | **Crude**  **OR** | **95% CI** | **P value** |
| --- | --- | --- | --- | --- | --- | --- | --- | --- | --- |
|  | **N=17** | **%** | **N=4** | **%** | **N=13** | **%** |  |  |  |
| Abnormal CT scan |  |  |  |  |  |  | 0.75 | 0.06-9.62 | 0.82 |
| No | 12 | 70.6 | 3 | 75.0 | 9 | 69.2 |  |  |  |
| Yes | 5 | 29.4 | 1 | 25.0 | 4 | 30.8 |  |  |  |
| Abnormal MRI |  |  |  |  |  |  | 4 | 0.19-84.2 | 0.37 |
| No | 15 | 88.2 | 3 | 75.0 | 12 | 92.3 |  |  |  |
| Yes | 2 | 11.8 | 1 | 25.0 | 1 | 7.7 |  |  |  |
| Abnormal EEG |  |  |  |  |  |  |  |  |  |
| No | 14 | 82.4 | 4 | 100.0 | 10 | 76.9 |  |  |  |
| Yes | 3 | 17.6 | 0 | 0.0 | 3 | 23.1 |  |  |  |
| Abnormal fundoscopic examination |  |  |  |  |  |  |  |  |  |
| No | 17 | 100 | 4 | 100.0 | 13 | 100.0 |  |  |  |
| Yes | 0 | 0.0 | 0 | 0.0 | 0 | 0.0 |  |  |  |
| EM suggested |  |  |  |  |  |  |  |  |  |
| No | 2 | 11.8 | 0 | 0.0 | 2 | 15.4 |  |  |  |
| Yes | 15 | 88.2 | 4 | 100.0 | 11 | 84.6 |  |  |  |

**Table 11: Treatment of New Caledonian cases of angiostrongyliasis according to death/sequelae, n=17**

| **Variables** | **Total** | | **Severe** | | | **Not severe** | | **Crude OR** | **95% CI** | | **P value** |
| --- | --- | --- | --- | --- | --- | --- | --- | --- | --- | --- | --- |
|  | **N=17** | **%** | **N=4** | | **%** | **N=13** | **%** |  |  |  |  |
| Albendazole |  |  | |  |  |  |  | 0.75 | 0.06-9.62 | 0.82 | |
| No | 12 | 70.6 | | 3 | 75.0 | 9 | 69.2 |  |  |  | |
| Yes | 5 | 29.4 | | 1 | 25.0 | 4 | 30.8 |  |  |  | |
| Length albendazole (d) | |  | |  |  |  |  |  |  | 0.39 | |
| Mean | 3.9 |  | | 6 (7.3) |  | 3.2 (1.1) |  |  |  |  | |
| Median | 3 |  | | 3 (0) |  | 3 (0) |  |  |  |  | |
| Stromectol |  |  | |  |  |  |  |  |  |  | |
| No | 16 | 94.1 | | 4 | 100.0 | 12 | 92.3 |  |  |  | |
| Yes | 1 | 5.9 | | 0 | 0.0 | 1 | 7.7 |  |  |  | |
| Length stromectol (d) | |  | |  |  |  |  |  |  | 0.7 | |
| Mean | 2.8 |  | | 2(0) |  | 3 (2.8) |  |  |  |  | |
| Median | 2 |  | | 2(0) |  | 2 (3) |  |  |  |  | |
| Steroids |  |  | |  |  |  |  | 16.5 | 1.09-250.2 | 0.04 | |
| No | 12 | 70.6 | | 1 | 75.0 | 11 | 84.6 |  |  |  | |
| Yes | 5 | 29.4 | | 3 | 25.0 | 2 | 15.4 |  |  |  | |
| Length steroids (d) |  |  | |  |  |  |  | 0.92 |  | 0.12 | |
| Mean | 33.5 |  | | 36 (47) |  | 31 (51) |  |  |  |  | |
| Median | 9 |  | | 15 (44) |  | 3 (45) |  |  |  |  | |
| Subtractive lumbar puncture |  |  | |  |  |  |  |  |  |  | |
| No | 16 | 94.1 | | 4 | 100.0 | 12 | 92.3 |  |  |  | |
| Yes | 1 | 5.9 | | 0 | 0.0 | 1 | 7.7 |  |  |  | |
| Antibiotics |  |  | |  |  |  |  |  |  |  | |
| No | 15 | 88.2 | | 4 | 100.0 | 11 | 84.6 |  |  |  | |
| Yes | 2 | 11.8 | | 0 | 0.0 | 2 | 15.4 |  |  |  | |

**Table 12: Final model of New Caledonian cases of angiostrongyliasis according to death/sequelae, n=17**

| **Variables** | **Total** | | **Death/sequelae** | | | **Without sequelae** | | **Adjusted OR** | | **95% CI** | | | **P value** |
| --- | --- | --- | --- | --- | --- | --- | --- | --- | --- | --- | --- | --- | --- |
|  | **N=17** | **%** | **N=4** | | **%** | **N=13** | **%** |  |  |  |  |  |  |
| Radiculitis |  |  | |  |  |  |  | 1.7 | | 1.25-2.5 | | 0.007 | |
| No | 13 | 76.5 | | 1 | 25.0 | 12 | 92.3 |  | |  | |  | |
| Yes | 4 | 23.5 | | 3 | 75.0 | 1 | 7.7 |  | |  | |  | |
| Positive serology in blood | |  | |  |  |  |  | 0.8 | | 0.58-1.2 | | 0.3 | |
| No | 13 | 76.5 | | 2 | 50.0 | 11 | 84.6 |  |  | |  |  |  |
| Yes | 4 | 23.5 | | 2 | 50.0 | 2 | 15.4 |  |  | |  |  |  |
| No | 13 | 76.5 | | 2 | 50.0 | 11 | 84.6 |  |  | |  |  |  |
| Modified MRI |  |  | |  |  |  |  | 0.6 | | 0.3-1.2 | | 0.2 | |
| No | 15 | 88.2 | | 3 | 75.0 | 12 | 92.3 |  | |  | |  | |
| Yes | 2 | 11.8 | | 1 | 25.0 | 1 | 7.7 |  | |  | |  | |
| Steroids |  |  | |  |  |  |  | 1.7 | | 1.16-2.6 | | 0.02 | |
| No | 12 | 70.6 | | 1 | 75.0 | 11 | 84.6 |  | |  | |  | |
| Yes | 5 | 29.4 | | 3 | 25.0 | 2 | 15.4 |  | |  | |  | |
